# First-Hand Recommendations for Nursing Management to Support Nurses Involved in the Process of Hastened Death: A Systematic Review of the Qualitative Evidence

**DOI:** 10.1155/2023/8601814

**Published:** 2023-05-11

**Authors:** Andrea Egger-Rainer, Sarah Bublitz, Stefan Lorenzl, Christiane Weck, Piret Paal

**Affiliations:** Institute of Palliative Care, Paracelsus Medical University, Salzburg, Austria

## Abstract

**Aim:**

The aim of this review was to support nursing management in creating frameworks for the care of people requesting hastened death, based on the best available evidence on the experiences of nurses.

**Background:**

The legalisation of hastened death presents nurses with a complex set of ethical and moral risks. The largely unregulated role of nurses in the politics of hastened death can lead to moral distress and burnout. *Evaluation*. A systematic database search was conducted in CINAHL, LIVIVO, Medline, OVID, and Web of Science. The meta-aggregative approach was used to synthesise the findings. Quality appraisal was done using criteria of the Joanna Briggs Institute. *Key Issues*. Sixteen studies from four different countries were included. Interview data were from 200 nurses. Meta-aggregation resulted in ten synthesised findings including the need for guidelines; time resources; a supporting team; and professional, social, and personal skills.

**Conclusions:**

A working environment with clear guidelines, sufficient resources, structured professional adjustment programmes, and educational measures is supportive for nurses. *Implications for Nursing Management*. Nursing management should create a professional strategy, guidelines, promote good team culture, implement education, and training activities.

## 1. Introduction

The legalisation of hastened death has confronted nurses with complex ethical and moral challenges in determining their own level of involvement in this new task. Nurses may already encounter problems when caring for geriatric people. It has been argued that the fear of hastening death by increasing the dose of morphine raises ethical issues [1]. Uncoordinated voluntary abstention from food and drink can also be a source of moral distress [2]. Especially chronically ill people suffering from cancer, neurological diseases (mostly motor neuron diseases), or end-organ failure (mostly heart failure and lung disease) express the wish for hastened death. They fear a loss of autonomy and control, suffer from unbearable physical symptoms or fear a suffering from them in the future (e.g., pain and nausea), or they have had negative experiences with death and dying in the past [3]. Furthermore, old age, feeling lonely, and socioeconomic hardship are linked to the wish of hastened death [4]. Good end-of-life care is important for nurses to prevent distressing events [5]. Regarding hastened death, two approaches can be distinguished: (a) voluntary active euthanasia, i.e., the lethal drug is administered by a physician or, as in Canada, also by a nurse practitioner; (b) assisted suicide, i.e., the lethal drug is prescribed by a physician and the person who wishes to die takes the drug independently [6]. The preparation of the lethal medication can be done by a healthcare professional. Dying is recognised as a discursively constructed process in which medical-physical, psychological, emotional, social, and spiritual support is necessary [7]. From this perspective, caring for people who wish hastened death is less a monoprofessional and more a multiprofessional matter. Therefore, the voices of all professional groups, such as physicians, nurses, social workers, and chaplains, should be heard in making the appropriate decisions [8, 9]. However, hastened death is primarily seen as the responsibility of physicians and the role of other professionals is rarely addressed [7, 10].

## 2. Background

The well-being of nurses is essential for integrated and efficient health service delivery. When nurses are unable to provide care in accordance with their own values, this can lead to psychological and moral distress and lower quality of care. Staff retention is negatively affected [11, 12]. Moral distress is often related to poor communication between the nurse and the interprofessional team, and the inability to fulfil a person's last wishes [1]. To reduce the burden, nurse managers need to recognise and understand the problems and create a supportive environment [13]. A scoping review of the role of nurses in hastened death has shown that nurses perform various tasks across the process continuum, from initial care and accompaniment, to assessing the person's condition, assistance in dying, debriefing, coordination, and documentation. However, depending on the country, their role is not always defined in the current legislation, which may lead them to practice outside the legal framework [14]. The unregulated role of nurses in hastened death policies jeopardises the safety of nurses (and patients) and may contribute to moral distress and burnout [15]. Apart from the professional regulations, there is also a lack of qualified training for nurses on the topic of hastened death [8, 16, 17]. Systematic instructions are required to protect nurses, facilitate a safe working environment, and prevent morally questionable incidents. It is therefore necessary to define which tasks fall within the responsibility of nurses and how they must be prepared [18].

This systematic review of qualitative studies aims to support nursing management in creating a framework for the care of people requesting hastened death, based on the best available evidence on the experiences of nurses. On that basis, appropriate structures can be implemented, and education and training schedules can be designed. The authors of this review are physicians, nurse, and anthropologist collaborating in a palliative care research group. In order not to bias the results of the review, the authors reflected on their personal attitudes to the topic in advance and discussed them regularly within the team. The study was guided by the questions “What experiences do nurses report who have cared for people requesting hastened death? What do nurse managers need to consider when designing a supportive environment for nurses?”

## 3. Methods

The meta-aggregative approach of the Joanna Briggs Institute (JBI) was adopted, rooted in the philosophy of pragmatism [19]. It is assumed that the goal of research is practical utility and that the new knowledge serves as a support for practical action [20]. The identified studies were subjected to a quality check in order to obtain reliable results [21]. As these are recommendations for concrete actions, they are presented in an if-then structure [19]. The review was registered on the PROSPERO platform (CRD42022322736). The guidelines “Enhancing transparency in reporting the synthesis of qualitative research (ENTREQ)” were followed for reporting [22].

### 3.1. Inclusion and Exclusion Criteria

All types of qualitative studies were included if the study participants were nurses with experience in caring for people who wished hastened death, and if the article was peer-reviewed. We did not set a time limit on the publication date that could be included, but hastened death had to be legal at the time the study was conducted. Studies were excluded if they reported on nurses' attitudes towards hastened death or if participants were conscientious objectors. Due to limited resources for translation, articles had to be published in English or German. For further information, please see Supplementary Material Table 1.

### 3.2. Search Strategy

A comprehensive literature search was conducted from July 2021 to December 2021 and updated in April 2022. Databases searched included CINAHL, LIVIVO, Medline via PubMed, OVID, and Web of Science. The PICo mnemonic was used to define the criteria *population* “nurses,” *phenomenon of interest* “experiences,” and *context* “care of people requesting hastened death” [21]. In addition, search terms were used that related to the study design. The search terms were combined with Boolean operators, and truncation was applied where appropriate (see Supplementary Material Table 2). Citation tracking was performed in the reference lists of the included articles and in the identified systematic reviews.

### 3.3. Study Screening Methods

The identified records were imported into the literature management programme Citavi (© Swiss Academic Software GmbH) and duplicates were removed. After the screening of the title and abstract, which was carried out by one researcher, the remaining full texts were checked for eligibility by two independent researchers. Discrepancies were discussed with a member of the research team. Before a final decision on inclusion was made, the quality of the studies was assessed.

### 3.4. Quality Appraisal

The quality appraisal was carried out using the JBI Critical Appraisal Checklist for Qualitative Research [23]. To provide confidence in the results, the ConQual approach was used for scoring. The score is composed of the dependability and credibility ratings. If the dependability score is 4 or 5, the study score remains high. If the rating is 2 or 3, the study is downgraded by one level, and if the rating is 0 or 1, the study is downgraded by two levels. To assess credibility, the findings of the studies (= level 1 findings) are graded as “unequivocal,” meaning that the finding is beyond doubt, “equivocal,” meaning that the finding is not entirely clear, or “unsupported,” meaning that no supporting data could be found in the study results. If the synthesised finding (= level 3 finding) contains only unequivocal findings, its credibility is rated “high”. A mixture of unequivocal and equivocal findings leads to a downgrade of 1 level and only equivocal findings to 2 levels. A mixture of equivocal and unsupported findings or only unsupported findings result in a downgrade of 3 and 4 levels, respectively [24]. Two researchers independently assessed study quality and discussed differences to reach a consensus.

### 3.5. Data Extraction

Firstly, the general details of the studies were extracted and summarised in a table. This includes authors, year of publication, methodology, country, participants, and phenomena of interest. Secondly, the level 1 findings of the studies, i.e., “*verbatim extract(s) of the author's analytical interpretation of the results*” [21] (p. 183), and corresponding illustrations, such as direct quotes from study participants, were extracted in individual Word documents for each study separately. The result sections of the studies were read several times to become familiar with the results. Only those results that could be clearly attributed to nurses were included. To ensure accuracy, data extraction was performed by one researcher and cross-checked by a second.

### 3.6. Data Synthesis

Level 1 findings were imported into QCAmap analysis programme [25] to be categorised and synthesised. Categories (= level 2 findings) were developed based on at least two level 1 findings that were similar in meaning. Two or more similar level 2 findings were combined to form level 3 findings [21]. The synthesis process was carried out through consensus discussions within the review team. For this purpose, the results were summarised in a table, reviewed and discussed within the team for conclusiveness, selectivity, and delimitability.

## 4. Results

### 4.1. Study Selection Results

**Figure 1:**
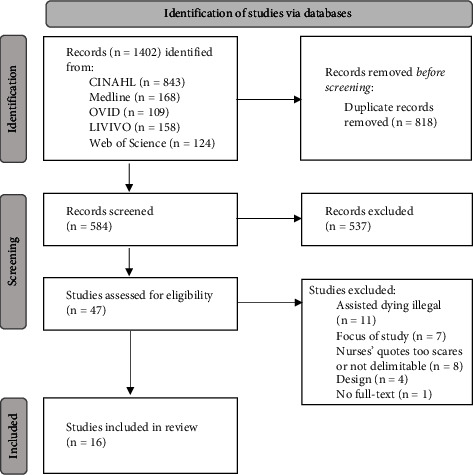
PRISMA flowchart describing the study selection process.

The Preferred Reporting Items for Systematic Reviews and Meta-Analysis (PRISMA) flowchart [26] in Figure 1 shows that 1402 studies were identified via databases. A total of 818 duplicates were removed and 537 records were excluded after title and abstract screening. The full texts of 47 studies were screened for eligibility. A total of 16 studies met the inclusion criteria.

### 4.2. Quality Appraisal Results (Studies/Dependability)

In 12 studies, the philosophical perspective remained unclear [27–38]. Five studies made a statement that situated the researchers culturally or theoretically [27, 35–37, 39]. The studies were of high methodological quality, except for four that were classified as “moderate” [28–30, 38]. In addition to the lack of indication of the philosophical perspective, the latter lack both a statement about the cultural or theoretical basis of the researchers and about their influence on the research. In two studies, the method of data collection did not fully match the methodology [28, 29], and in one study, the presentation of data was confusing [30]. Overall, the dependability scores of the studies ranged from 2 to 5 (see Table 1).

### 4.3. Study Characteristics

The 16 studies were conducted in Belgium (*n* = 5), Canada (*n* = 9), Switzerland (*n* = 1), and The Netherlands (*n* = 1). They were published between 1998 and 2021 and used different methodological approaches: Grounded Theory (*n* = 6), interpretive description (*n* = 3), narrative enquiry (*n* = 2), in-depth qualitative enquiry (*n* = 1), qualitative description (*n* = 1), qualitative interview (*n* = 1), and qualitative unspecified enquiry (*n* = 2). A total of 200 nurses were interviewed, with interviews with 104 nurses analysed in more than one study. The methods of analysis used were thematic analysis (*n* = 6), grounded theory (*n* = 4), interpretive descriptive method (*n* = 3), constant comparative method (*n* = 2), and deductive categorisation (*n* = 1). Three studies examined nurses' involvement in hastened death. Three other studies aimed to determine the experiences of nurses in caring for people requesting hastened death. One study each examined the meaning of suffering, communication, moral experiences, and the experience of support in relation to hastened death. One study each looked at the care of people requesting hastened death in the context of palliative care and in the context of care for older people. Two studies each examined the role of nurses and the implications for nursing practise. The characteristics of the studies with references are summarised in Table 2.

### 4.4. Quality Appraisal Results (Findings/Credibility)

No distinction was made between voluntary active euthanasia and assisted suicide. Results from nurses who were legal providers or conscientious objectors were excluded. In some studies, the phenomenon of interest also included the experiences of other health professionals. Therefore, level 1 findings that were not supported by a direct quote from a nurse were rated equivocal. A total of 310 unequivocal and 124 equivocal findings were included and 40 unsupported findings were excluded. Many of the unsupported findings could also be found as supported findings in other studies and were therefore included in the synthesis in this way, such as overwhelming feelings, highly professional care, and importance of communication. However, there were also unsupported findings that had only been made in individual studies and could therefore not be included in the synthesis. This concerned, for example, statements about spatial difficulties when people did not want to carry out assisted suicide at home [40] or about informing the team about a person's expressed wish to die without having asked the person's permission beforehand [38]. The synthesised findings are considered valid, as the ConQual scores of six of them are classified as “moderate” and the ConQual scores of four as “high” (see Table 3).

### 4.5. Review Findings

The meta-aggregation produced ten synthesised findings. These comprise two, three, or four categories, respectively (see Supplementary Material Table 3).

#### 4.5.1. Synthesised Finding 1: If Nurses Are to Provide Good Care, They Need Sufficient Time Resources

Problems related to lack of resources comprise three categories: “care for patients requesting hastened death is time consuming,” “nurses face time restrictions,” and “time constraints may have consequences.” Nurses report a negative impact on the care of people requesting hastened death caused by a lack of resources, especially time (A4, A10, and A13), because *“time is of essence for a good experience of the process”* (A6, p. 44). It is important for nurses to provide good care (A6 and A8), but caring for people who wish hastened death is time consuming and can be at the expense of caring for others (A9 and A10). Nurses need time to connect with the person to build a relationship and better understand the request (A1, A13, and A15):

  The man came to the hospital for his euthanasia, and you don't know him, and he only came for his euthanasia. (…) We weren't involved in any way, and I felt horrible about it, like, he comes over to die, and we haven't had any conversation with him, we didn't know him. (A1, p. 499)

#### 4.5.2. Synthesised Finding 2: If Nurses Are to Provide Good Care, They Need a Supporting Team

The importance of a well-functioning team is described in three categories: “being part of a team and cooperation within a team is essential,” “debriefings are helpful to overcome uncertainty,” and “senior nurses and managers play an important role for functioning teams.” Team culture is critical to how nurses experience hastened death and whether or not they feel judged by team members (A11). The quality of collaboration within the interprofessional team determines the quality of the process (A1, A2, A6, A7, and A13). It is the responsibility of senior nurses to promote good group dynamics (A8), as different attitudes among team members can cause problems (A9 and A11). While conscientious objectors may feel excluded from caring for people requesting hastened death (A15), supporters may see resistance from their colleagues as a barrier to good care. Supporters may also feel isolated from the team, especially if managers appear to be among the objectors (A11). But team members learn to respect different attitudes (A16) and even often opposing views of palliative care nurses and hastened death supporters can develop positively (A13). When well-functioning teams have formed, it can be difficult for newcomers to be included and gain experience (A13). It is considered important to be part of a team and be able to help and support each other (A2, A12–A14). Senior nurses try *“to be there as a pillar for the team, someone they can rely on*” (A7, p. 3376). In doing so, they feel responsible for both the nurses and the physicians (A8). Formal debriefings, either at the individual or team level, are considered helpful (A9 and A16). Informal conversations cannot replace these formal debriefings (A11), although a *“conversation in the corridor”* (A16, p. 504) can be a source of support (A11 and A16). However, nurses may also feel unsupported in conversation, which can be accompanied by insensitive behaviour (A11). Nurse managers need to facilitate communication and support teams to create new forms of rituals: *“We needed some sort of ceremony around it because it felt so weird because there wasn't any preamble or post anything”* (A15, p. 7).

#### 4.5.3. Synthesised Finding 3: If Nurses Are to Provide Good Care, They Need Clear Guidelines and Policies

This finding reports on the need for a clear definition of responsibilities and processes. It includes two categories: “the caring process is complex, needs good organisation and develops over time” and “clear guidelines and policies help to reduce uncertainty.” The care process for people requesting hastened death has been described as complex and dynamic (A5, A7, A8, and A13). It can be compromised by inadequate teamwork and the care system (A4 and A13). Accurate planning, good organisation, and early identification of potential problems are crucial for high process quality (A5, A7, A8, and A15). The lack of clear guidelines can lead to uncertainty and make nurses uncomfortable (A1, A2, A4, A5, A13, and A15). Then, they may even be unsure whether they are allowed to talk about hastened death (A15). Moreover, *“introducing the topic* (…) *might be misinterpreted as an invitation to request for it”* (A10, p. 450). Practical aids, e.g., care protocols and checklists, proved helpful (A5, A15, and A13). Over time, the process evolves, and care becomes easier. The team moves forward and feels prepared for future requests (A5–A7, A10, A13, and A14).

#### 4.5.4. Synthesised Finding 4: If Nurses Are to Provide Good Care, They Need Professional Skills

There is a need for qualified nurses with high professional expertise, comprising three categories: “caring for persons requesting hastened death is not routine nursing care and may lead to professional satisfaction,” “nurses must learn to distinguish between professional role and personal views,” and “there is a need for further training.” A high demand for professional nursing skills has been described, ranging from understanding suffering to postmortem activities (A2, A12, and A15). Care for people who wish hastened death is considered part of holistic care (A2, A4, A7, A15, and A16), where nurses need to provide the best possible care (A1, A5–A8, A13, and A16) to achieve *“the most patient-centered death possible”* (A15, p. 7). If hastened death is a new field, nurses may face the challenge of generating new knowledge by themselves (A15). This can be seen as an opportunity to change routine nursing practice (A1, A2, A5, A12, A14, and A15). It may also lead to a change in the understanding and scope of nursing (A4, A14, and A16). Although it is not always easy, nurses try to separate their personal views from their professional role (A5, A6, A13, and A15): *“And then I tell myself: “Come on, X, it is not about what you feel, it is about being there for the patient, for the family.” […] I really can separate the two”* (A6, p. 43). Some nurses report professional fulfilment and satisfaction due to their role in the hastened death process (A1–A3 and A10) and a possible change in their career, which can be positive or negative (A14). However, nurses lack training (A2 and A15). They are concerned that *“lack of mandatory education might contribute to routinizing death and cause unintended harm”* (A3, p. 273) and suggest specific education and training (A9).

#### 4.5.5. Synthesised Finding 5: If Nurses Are to Provide Good Care, They Need Personal Skills

There is a need for qualified nurses with competencies at a personal level, firstly, “nurses have to respect the person and her/his decision” and secondly, “nurses see themselves confronted with ethical challenges.” Nurses emphasise that it is important to respect the person and her/his wish to die without judging (A2, A4, A5, A8, and A14). Because they value the relationship with the person, nurses accompany her/him on this journey. Life should end well (A4 and A5). It is easier if the person is absolutely sure about the decision or if the nurses can understand the wish to die (A14 and A16): *“With this man I thought: you are right, I would make the same decision…I could empathize with what he wanted”* (A16, p. 501). Although legal and in line with the person's wishes, hastened death can be experienced as an ethical challenge and accompanied by moral uncertainty (A9, A10, and A14): *“At the beginning I thought having more options, the better for people. What I'm realising is that sometimes having that option* (…) *causes suffering for the person passing away, for their loved ones, and for healthcare providers in general'*' (A9, p. 191). To support people asking for hastened death and their families, nurses need to be open, attentive, and trustworthy. They need to reflect on their own attitudes towards death and learn to talk about it (A3 and A7):

  I think we're gonna do much better if we start the conversation early in our training to be more prepared to talk about these things. (…) So I think exposure to conversation about (death and dying) and you know, at least bringing some attention to it … can make this process a lot more fluid for all concerned. Because it's not just the patient. (A3, p. 274)

#### 4.5.6. Synthesised Finding 6: If Nurses Are to Provide Good Care, They Need Social Skills

The need for qualified nurses with competencies at a social level contains four categories: “nurses must be able to establish relationships;” “nurses act as advocates, guides, and supporters;” “nurses must provide information;” and “nurses need communication competencies.” It is important for nurses to quickly establish a relationship with the person requesting hastened death and family members (A5, A12, and A15). Advocacy is the key (A7, A8, and A12). Nurses describe themselves as supporters and guides through the process (A5, A7, A15, and A16). They keep in mind what might be unexpected for those involved, while always prioritising the person's wishes (A7, A9, and A15): *“I think one of the biggest roles we can be as nurses are advocates, and advocating for what your patient wants is not necessarily what you would want for yourself”* (A12). If they have not been involved in the decision-making process by the person requesting hastened death, nurses critically question preexisting relationships, for example, in nursing homes or palliative care (A4, A10, and A13): *“Me, I struggled because I was wondering why she had ended up in such a situation. Was it us, had we not done enough?”* (A4, p. 607). Thorough information about end-of-life options, including palliative care alternatives, is important for people to make the right decision (A2, A5, A7, and A14). An exit strategy should be in place in case they change their mind (A15). Sometimes it can be difficult for nurses to fully understand the request (A1, A5, A6, and A16). As people often use vague terms (A16), nurses need to thoroughly assess individual values and expectations (A7, A15, and A16). It is necessary to reflect on the possible reasons for the wish to die (A7 and A15):

  When we have a patient who says, loud and clearly: “want euthanasia! Don't bring me all the twaddle of palliative care. I want euthanasia!” Even then, we […] say: “Okay, we want to have these conversations with you. We want to listen. (…) Just to make sure that it is your wish to die instead of merely wanting to be rid of physical pain” (A7, pp. 3375–3376).

If nurses are open minded, alert, and attentive, the request should not come as a surprise (A8). Nurses emphasise the importance of communication with all members of the interprofessional team (A1, A2, and A7). It is important that the team is well informed (A5, A7, and A16), that each team member has the opportunity to contribute to the discussion and that they actively listen to each other (A7 and A8). Nurses should be actively involved in decision-making because of their close relationship to the person wishing to die—but this is not always the case (A6, A8, and A16): *“*(…) *we sometimes have the feeling that the doctor decides fully independently (from the nursing team).* (…) *This is such a pity, because we do build up a different sort of relationship with the patient than the physician does”* (A6, p. 44).

When talking to people requesting hastened death and families, it is important to create a communicational atmosphere, to take time to listen attentively and to address issues that are not related to hastened death. Nurses need to learn what words are appropriate and how to address a request (A1, A5, A7, A9, A10, and A15): *“If you miscommunicate and impair your relationship with your patient, they may not trust you with this kind of sensitive information. We should have clinicians practice with simulated patients, and experts in communication can give feedback”* (A9, p. 192). Throughout the process, nurses should *“simply be there”* (A7, p. 3375) for the person and family (A7 and A12) and, at the time of action, support *“anyone who needs it (patient, family, colleagues, physician)*” (A8, p. 2415). To *“create closure in time and space”* (A5, p. 270), nurses say goodbye to the dying person, take care of the family, and stay in contact with the family beyond that, e.g., by attending the funeral (A5, A8, A9, A15, and A16).

#### 4.5.7. Synthesised Finding 7: If Nurses Are to Provide Good Care, They Need to Change Their Perspective

Nurses need to rethink their current perspective: “the experience of a hastened death may differ from that of a natural death,” “nurses understanding of the value of life may differ from that of the person requesting hastened death,” and “nurses may be insecure about the request.” Nurses need to reflect on their own attitudes towards hastened death. This can be particularly challenging if their understanding of the value of life differs from that of people requesting hastened death (A1 and A2). With increasing experience, nurses' view of dying may change (A3). As a planned phenomenon (A1, A3, and A12), this form of death is often experienced as more peaceful and deliberate than natural death (A6) and sometimes has a celebratory character (A2 and A12). It may seem unreal how quickly death occurs (A15 and A16):

  The process is so incredibly smooth and peaceful that there's no trauma involved in watching it happen. […] The patient literally says yeah, I feel. . . and they're asleep. And then the stuff that ends their life happens while they're sound asleep. It's really incredibly peaceful. I think the whole thing takes something like five minutes, and they're asleep after the first 30 seconds. (A12)

When patients die, nurses can be emotionally affected on a personal level (A4). While most nurses perceive hastened death and natural death as similar, others state that the natural death of people they have known for a long time can be even more difficult (A8 and A11). For some, hastened death is a violent form of death (A4) and a negative experience, especially when relatives cannot accept the person's decision (A10). They are aware of a potential misuse (A3, A9, and A16).

#### 4.5.8. Synthesised Finding 8: If Nurses Are to Provide Good Care, They Must Act as Mediators

Nurses are torn between two or more parties, such as “…between the person requesting hastened death and the family” and “…between the person requesting hastened death and the interprofessional team.” When disagreements arise between family and the person requesting hastened death, nurses act as mediators, trying to make peace and resolve tensions (A7, A8, and A10). In the interprofessional team, nurses try to evoke an understanding of the person's decision (A7 and A12). While the request should be discussed between the person and physician (A16), nurses act as translators to ensure that both parties understand each other correctly (A7). Difficulties can arise when physicians do not stick to agreements and nurses are not allowed to answer questions from the person requesting hastened death (A6):

  So that (the palliative sedation) in itself went well and all, but it has not been the patient's wish. We were not acting in a correct manner. The request had been uttered before. (…) And then it becomes difficult, because the doctor comes and goes, but as a nurse you provide 24h-care. (A6, pp. 44–45)

#### 4.5.9. Synthesised Finding 9: If Nurses Are to Provide Good Care, They Need to Know that There Will be Emotional Ups and Downs

Several emotional challenges that can be associated with this task, such as “caring for persons requesting hastened death is emotionally demanding and may come along with positive and negative feelings,” “nurses need help when deciding for or against participating in hastened death,” and “nurses must develop coping strategies.” Nurses feel a great sense of responsibility and describe caring for people requesting hastened death as demanding, intense, upsetting, and exhausting (A1, A4, A6, A10, and A14–A16). Caregiving can be associated with feelings of sadness, distress, and frustration (A1, A2, A9, and A14) and with a *“yet unknown emotional impact over time of aiding in assisted deaths”* (A3, p. 273). Some nurses think about it for several weeks after a hastened death (A4) and feel they can never get used to it (A6). Coping strategies such as a task-oriented approach, physical activities, and talking with the partner can be helpful (A3, A6, and A14). Some nurses also report a mixture of conflicting emotions and surprisingly positive feelings (A1, A2, and A6). They experience working in this field as a personal growth process (A5) and describe it as satisfying. They feel happy and grateful to be able to help (A2–A4, A6, A12, and A14). They also feel it is an honour and privilege to share this intimate moment with someone (A1 and A2) from whom they receive gratitude (A3 and A14):

  The patients always say that they're grateful that their suffering is going to be over and the family members, whether they agree with the care option or not, because a lot of family members think this is awful, even after the assisted death has happened, the family members usually say, I'm happy that their suffering's over. (A14, p. 3879)

#### 4.5.10. Synthesised Finding 10: If Nurses Are to Provide Good Care, They Need to Know that Hastened Death Will Affect Palliative Care

Hastened death may impact providing and receiving of palliative care. The aspects were pointed out: “nurses must be aware that hastened death is a different approach to natural death,” “nurses struggle with structural shortcomings,” and “nurses observe conflicts between palliative care and hastened death.” Nurses report that the legalisation of hastened death could have a negative impact on palliative care. On the one hand, they fear that people believe that palliative care will accelerate death (A9 and A13). On the other hand, they observe that dying naturally is less accepted and people insist on a quick death (A1 and A6). While waiting for hastened death, it can be difficult for nurses to maintain the eligibility of the person and pain and symptom control (A10 and A13). Some nurses are certain that palliative care could have provided good solutions in many cases, but people are unwilling to compromise or have difficulty accessing palliative care services (A13 and A15). It is problematic when the media focuses more on hastened death (A9) and resources are shifted from palliative care to this form of dying (A10).

## 5. Discussion

This review summarises the findings of 16 studies on nurses' experiences of caring for people requesting hastened death. The legalisation of hastened death confronts nurses with a complex set of ethical and moral risks. The review has shown that nurses need to know what they are allowed to do, they need to have the appropriate skills and sufficient time. Although a person's wish is followed, caring for persons requesting hastened death is described as ethically challenging due to its high complexity. Of particular concern are the potential long-term personal and professional effects on nurses' emotions and moral values, which may affect their ability to work. Nurse managers need to create a supporting environment to prevent nurses from moral distress and burnout, including providing opportunities for moral deliberation and supervision.

Guidelines clearly outlining nursing competencies in relation to hastened death are therefore necessary [8, 16]. The WHO [18] stresses the importance of competency frameworks that are fully applied in nursing practice and that nurses are involved in the development of these guidelines. The nursing process for people requesting hastened death begins with the request and does not end with the death of the person, but still includes the care of the grieving relatives. Nurses need to be recognised as an essential part of the decision-making process by law and by the interprofessional team. If this is the case, they can understand and accept why a hastened death is being carried out [16].

Caring for people requesting hastened death is emotionally demanding and challenges personal values and beliefs. The well-being of nurses requires structures where formal and informal psychosocial and spiritual support is available [6, 18, 43]. Debriefing provides an opportunity to talk openly about difficult situations and allows nurses to reflect on their experiences. Educational needs can be identified. This improves the process and can protect professionals from burnout [44,45]. Time is essential to build relationships with the person who wishes to die and the family, and not having enough time can be perceived as a moral failure [6]. Therefore, safe staffing is necessary [18], i.e., there must be enough nurses on duty at all times [46]. Senior nurses need leadership skills to ensure a good team culture in well-functioning interprofessional teams. Different attitudes within the team must be accepted [43] and should not cause conflict [47]. Nurses need support when they have to decide whether to participate in hastened death or not. Their decision must be accepted without condemnation. All team members need to be aware that there may be different reasons for the decision and that attitude towards participation can change. New nurses need to be welcomed by the established team to enable them to gain experience themselves. This process can be supported by a buddy system that promotes both onboarding and knowledge sharing. In a buddy system, an experienced nurse accompanies the new nurse through the first weeks on the ward. If new team members have fixed reference persons, they can familiarise themselves with the unit culture more quickly and feel comfortable [48].

The review has shown that specific education for all nurses should be introduced. Preparation for *“this new type of death”* [49] (p. 227) is essential, and nurses need to learn to reflect on their own attitudes towards (assisted) dying and death. Training should be provided by experienced nurses [18]. As these are rare in this field, the development of online programmes, such as Massive Open Online Courses (MOOCs), should be considered. Thereby, nurses could benefit from the knowledge of experts, regardless of location [50].

Studies in this review emphasise the importance of communication skills. As in previous studies, it was found that nurses need to learn how to respond to a request and how to determine if hastened death is really what the person wants [43,51]. Simulation training to practice communication is advisable [18,52]. The research-based theatre method could be used to increase empathy and promote interprofessional collaboration. Nurses would have the opportunity to put themselves in different roles to better understand the feelings of the person affected. They would also be able to reflect on their own role in the nursing process and the challenges they may face when caring for these people [53]. Similarly, training in palliative and end-of-life care should be provided. Nurses need to learn to explore and address untreated suffering [16,43] and be competent in managing pain and symptoms at the end-of-life.

### 5.1. Limitations

There are different legal regimes for hastened death in different countries. They differ in the location where the dying process is carried out (e.g., at home, in the hospital, and in a special facility), in access requirements, and in who may provide assistance in dying and in what way. However, we were unable to make any distinctions here, with the exception that we excluded quotes from nurse providers. Little information was provided about the demographic characteristics of nurses, such as ethnicity and religious affiliation that might influence their decision-making and experiences. The 16 articles were from nine studies conducted in four countries. Although hastened death is legal in several countries and states of the United States of America (USA), no other suitable studies were found. This may be due to the fact that in most of these countries, the law has only recently been enforced. In the US-studies, the results of interviews with different health professionals were published together, and the quotes of nurses were either too few or the experiences of nurses were not delineated. It must therefore be assumed that the scope of experience is limited.

## 6. Conclusions

Care of people requesting hastened death can be challenging for nurses. A supportive environment can be helpful to avoid moral distress and burnout. In most countries, the care of people requesting hastened death is a new field in nursing practice. As society changes, this field will also change, experience and knowledge will increase. In the words of one nurse: *“It's like living grounded theory. We're making it up as we go along”* [40] (p. 8). Continued research on this topic is essential to inform policy makers, professional associations, and nurses in management and practice.

## 7. Implications for Nursing Management

To avoid moral distress and burnout of nurses when caring for people requesting hastened death, they need a supportive environment. Therefore, nurse managers should create frameworks in terms of (1) establishing guidelines that address the role of nurses in hastened death, (2) create a professional strategy in responding to requests for hastened death, (3) providing sufficient (time) resources, (4) establishing regular debriefings, (5) promoting a good team culture, (6) introducing a structured professional adjustment programme for new members of the nursing team, and (7) integrating the topic of hastened death into education and training.

## Figures and Tables

**Table 1:** Appraisal of study quality and assessment of dependability.

Study	Q1	Q2	Q3	Q4	Q5	Q6	Q7	Q8	Q9	Q10	Dependability score
Bellens et al. [27]	U	Y	Y	Y	Y	Y	Y	Y	Y	Y	5
Beuthin et al. [28]	U	Y	N	Y	U	N	N	Y	Y	Y	2
Bruce and Beuthin [29]	U	Y	N	Y	U	N	N	N	Y	Y	2
Castelli Dransart et al. [30]	U	Y	Y	U	Y	N	N	Y	Y	Y	2
Denier et al. [31]	U	Y	Y	Y	Y	N	Y	Y	Y	Y	4
Denier et al. [32]	U	Y	Y	Y	Y	N	Y	Y	Y	Y	4
Denier et al. [33]	U	Y	Y	Y	Y	N	Y	Y	Y	Y	4
Dierckx de Casterlé et al. [34]	U	Y	Y	Y	Y	N	Y	Y	Y	Y	4
Ho et al. [35]	U	Y	Y	Y	Y	Y	N	Y	Y	Y	4
Mathews et al. [39]	Y	Y	Y	Y	Y	Y	Y	U	Y	Y	5
Mills et al. [36]	U	Y	Y	Y	Y	Y	Y	Y	Y	Y	5
Mills et al. [37]	U	Y	Y	Y	Y	Y	Y	U	Y	Y	5
Pesut et al. [40]	Y	Y	Y	Y	Y	N	Y	Y	Y	Y	4
Pesut et al. [41]	Y	Y	Y	Y	Y	N	Y	Y	Y	Y	4
Pesut, Thorne Tishelman [42]	Y	Y	Y	Y	Y	N	Y	Y	Y	Y	4
van de Scheur and van der Arend [38]	U	Y	Y	Y	Y	N	N	Y	Y	Y	3

Note: Y = yes; N = no; U = unclear; dependability scores derived from Q2, 3, 4, 6, and 7. JBI critical appraisal checklist for qualitative research [23]. Q1: Is there congruity between the stated philosophical perspective and the research methodology? Q2: Is there congruity between the research methodology and the research question or objectives? Q3: Is there congruity between the research methodology and the methods used to collect data? Q4: Is there congruity between the research methodology and the representation and analysis of data? Q5: Is there congruity between the research methodology and the interpretation of results? Q6: Is there a statement locating the researcher culturally or theoretically? Q7: Is the influence of the researcher on the research, and vice versa, addressed? Q8: Are participants, and their voices, adequately represented? Q9: Is the research ethical according to current criteria or, for recent studies, and is there evidence of ethical approval by an appropriate body? Q10: Do the conclusions drawn in the research report flow from the analysis, or interpretation, of the data?

**Table 2:** Study characteristics.

Study year of publication		Methodology	Data collection	Country	Participants	Phenomena of interest
			Data analysis			
A1	Bellens et al. [27]	Grounded theory	Semistructured interviews	Belgium	26 nurses	How nurses experience their involvement in the care of patients requesting euthanasia
			Constant comparison method			
A2	Beuthin et al. [28]	Narrative enquiry approach	Semistructured interviews	Canada	17 nurses	Nurses' experience of either providing care for a patient who had chosen MAiD, or declining to participate in MAiD
			Thematic analysis			
A3	Bruce and Beuthin [29]	Narrative enquiry approach	Semistructured interviews	Canada	17 nurses	How nurses' experiences of suffering are being shaped through caring for patients and families choosing MAiD
			Thematic analysis			
A4	Castelli Dransart et al. [30]	Grounded Theory	Semidirective interviews	Switzerland	28 nurses	Stances of health and social care professionals confronted with requests for assisted suicide from older people
			Constant comparison method		12 HCPs	
A5	Denier et al. [31]	Grounded Theory	In-depth interviews	Belgium	18 nurses	Nurses' involvement in caring for patients requesting euthanasia
			Grounded Theory			
A6	Denier et al. [32]	Grounded Theory	In-depth interviews	Belgium	18 nurses	Nurses' experience in caring for patients requesting euthanasia
			Grounded Theory			
A7	Denier et al. [33]	Grounded Theory	In-depth interviews	Belgium	18 nurses	Nurses' perspectives on communication during the euthanasia care process
			Grounded Theory			
A8	Dierckx de Casterlé et al. [34]	Grounded Theory	In-depth interviews	Belgium	18 nurses	Nurses' involvement in the care process for patients requesting euthanasia
			Grounded Theory			
A9	Ho et al. [35]	Qualitative interview study	Semistructured interviews	Canada	12 nurses	Palliative health care professionals' experiences and perspectives in providing care after the legalisation of MaiD
			Thematic analysis		14 HCPs	
A10	Mathews et al. [39]	Qualitative descriptive	Semistructured interviews	Canada	10 nurses	Impact of MAiD on palliative care practice for physicians and nurses in Canada
			Thematic analysis		13 HCPs	
A11	Mills et al. [36]	Qualitative unspecified	Semistructured interviews	Canada	10 nurses	Experience of support from the perspective of staff directly involved in the care of patients asking about or receiving MAiD
			Thematic analysis		11 HCPs	
A12	Mills et al. [37]	In-depth qualitative study	Semistructured interviews	Canada	10 nurses	How health care professionals perceive their roles as care providers; explore their reasons for viewing or not viewing MAiD as care
			Thematic analysis		11 HCPs	
A13	Pesut et al. [40]	Interpretive description	Semistructured telephone interviews	Canada	59 nurses	How nurses construct good nursing practice within the context of MAiD
			Interpretive descriptive method			
A14	Pesut et al. [41]	Interpretive description	Semistructured telephone interviews	Canada	59 nurses	Nurses' moral experiences with MAiD in the Canadian context
			Interpretive descriptive method			
A15	Pesut et al. [42]	Interpretive description	Semistructured telephone interview	Canada	59 nurses	Understand the implications of a legislated approach to assisted death for nurses' experiences and nursing practice
			Interpretive descriptive method			
A16	van de Scheur and van der Arend, [38]	Qualitative unspecified	Semistructured in-depth interviews	The Netherlands	20 nurses	Describe the role of nurses in euthanasia
			Deductive categorisation			

Note: HCP = health care professional; MAiD = medical assistance in dying.

**Table 3:** ConQual scores of synthesised findings.

Synthesised finding	Type of research	Dependability^†^	Credibility^‡^	ConQual score
If nurses are to provide good care, they need sufficient time resources	Qualitative	High	Downgrade 1 level	Moderate
If nurses are to provide good care, they need a supporting team	Qualitative	High	High	High
If nurses are to provide good care, they need clear guidelines and policies	Qualitative	High	High	High
If nurses are to provide good care, they need professional skills	Qualitative	High	Downgrade 1 level	Moderate
If nurses are to provide good care, they need personal skills	Qualitative	High	High	High
If nurses are to provide good care, they need social skills	Qualitative	High	Downgrade 1 level	Moderate
If nurses are to provide good care, they need to change their perspective	Qualitative	High	Downgrade 1 level	Moderate
If nurses are to provide good care, they must act as mediators	Qualitative	High	Downgrade 1 level	Moderate
If nurses are to provide good care, they need to know that there will be emotional ups and downs	Qualitative	High	High	High
If nurses are to provide good care, they need to know that hastened death will affect palliative care	Qualitative	High	Downgrade 1 level	Moderate

Note: ^†^High: the dependability score of more than 50% of the contributing studies is 4 or 5. ^‡^High: the synthesised finding contains only unequivocal findings. Downgrade 1 level: the synthesised finding contains a mixture of unequivocal and equivocal findings.

## Data Availability

No data were used to support the findings of this study.

## Supplementary Materials

Supplementary Materials Supplementary Table 1: inclusion and exclusion criteria. Supplementary Table 2: search strategy. Supplementary Table 3: synthesised findings, categories, findings, and corresponding illustrations.

## References

[1] Piers R. D., Versluys K., Devoghel J., Vyt A., van den Noortgate N. (2019). Interprofessional teamwork, quality of care and turnover intention in geriatric care: a cross-sectional study in 55 acute geriatric units. *International Journal of Nursing Studies*.

[2] Stängle S., Fringer A. (2022). Discussion or silent accompaniment: a grounded theory study about voluntary stopping of eating and drinking in Switzerland. *BMC Palliative Care*.

[3] Wiebe E., Shaw J., Green S., Trouton K., Kelly M. (2018). Reasons for requesting medical assistance in dying. *Canadian family physician Medecin de famille canadien*.

[4] Briggs S., Lindner R., Goldblatt M., Kapusta N., Teising M. (2022). Psychoanalytic understanding of the request for assisted suicide. *The International Journal of Psychoanalysis*.

[5] De Brasi E. L., Giannetta N., Ercolani S. (2021). Nurses’ moral distress in end-of-life care: a qualitative study. *Nursing Ethics*.

[6] Elmore J., Wright D. K., Paradis M. (2018). Nurses’ moral experiences of assisted death: a meta-synthesis of qualitative research. *Nursing Ethics*.

[7] Lang A. (2020). The good death and the institutionalisation of dying: an interpretive analysis of the Austrian discourse. *Social Science & Medicine*.

[8] Fujioka J. K., Mirza R. M., McDonald P. L., Klinger C. A. (2018). Implementation of medical assistance in dying: a scoping review of health care providers’ perspectives. *Journal of Pain and Symptom Management*.

[9] Roest B., Leget C. (2022). A multidisciplinary perspective on physician-assisted dying in primary care in The Netherlands: a narrative interview study. *Death Studies*.

[10] Cayetano-Penman J., Malik G., Whittall D. (2021). Nurses’ perceptions and attitudes about euthanasia: a scoping review. *Journal of Holistic Nursing*.

[11] Lamiani G., Borghi L., Argentero P. (2017). When healthcare professionals cannot do the right thing: a systematic review of moral distress and its correlates. *Journal of Health Psychology*.

[12] Trautmann J., Epstein E., Rovnyak V., Snyder A. (2015). Relationships among moral distress, level of practice independence, and intent to leave of nurse practitioners in emergency departments: results from a national survey. *Advanced Emergency Nursing Journal*.

[13] O’Donnell C., Markey K., O’Brien B. (2022). Guiding nurse managers in supporting nurses in dealing with the ethical challenge of caring. *Journal of Nursing Management*.

[14] Bellon F., Mateos J. T., Pastells-Peiró R., Espigares-Tribó G., Gea-Sánchez M., Rubinat-Arnaldo E. (2022). The role of nurses in euthanasia: a scoping review. *International Journal of Nursing Studies*.

[15] Weston M. (2010). Strategies for enhancing autonomy and control over nursing practice. *Online Journal of Issues in Nursing*.

[16] Busquets-Surribas M. (2021). The ethical relevance of nursing care in euthanasia and assisted suicide. *Enfermeria Clinica (English Edition)*.

[17] McMechan C., Bruce A., Beuthin R. (2019). Canadian Nursing Students’ Experiences with Medical Assistance in Dying/Les expériences d’étudiantes en sciences infirmières au regard de l’aide médicale à mourir. *Quality Advancement in Nursing Education - Avancées En Formation Infirmière*.

[18] World Health Organization [WHO] (2021). Building Better Together. Roadmap to Guide Implementation of the Global Strategic Directions for Nursing and Midwifery in the WHO European Region. https://apps.who.int/iris/bitstream/handle/10665/350207/WHO-EURO-2021-4464-44227-62471-eng.pdf.

[19] Hannes K., Lockwood C. (2011). Pragmatism as the philosophical foundation for the Joanna Briggs meta-aggregative approach to qualitative evidence synthesis. *Journal of Advanced Nursing*.

[20] Basile P., Basile P., Röd W. (2014). Der amerikanische Pragmatismus [American pragmatism]. *Die Philsophie des ausgehenden 19. und 20. Jahrhunderts 1: Pragmatismus und analytische Philosophie [The philosophy of the late 19th and 20th centuries 1: Pragmatism and analytical philosophy]*.

[21] Lockwood C., Munn Z., Porritt K. (2015). Qualitative research synthesis: methodological guidance for systematic reviewers utilizing meta-aggregation. *International Journal of Evidence-Based Healthcare*.

[22] Tong A., Flemming K., McInnes E., Oliver S., Craig J. (2012). Enhancing transparency in reporting the synthesis of qualitative research: ENTREQ. *BMC Medical Research Methodology*.

[23] Joanna Briggs Institute [JBI] (2017). Checklist for Qualitative Research. https://jbi.global/sites/default/files/2019-05/JBI_Critical_Appraisal-Checklist_for_Qualitative_Research2017_0.pdf.

[24] Munn Z., Porritt K., Lockwood C., Aromataris E., Pearson A. (2014). Establishing confidence in the output of qualitative research synthesis: the ConQual approach. *BMC Medical Research Methodology*.

[25] Fenzl T., Mayring P. (2017). QCAmap: eine interaktive Webapplikation für Qualitative Inhaltsanalyse [QCAmap: an interactive web application for Qualitative Content Analysis]. *Zeitschrift für Soziologie der Erziehung und Sozialisation*.

[26] Page M. J., McKenzie J. E., Bossuyt P. M. (2021). The PRISMA 2020 statement: an updated guideline for reporting systematic reviews. *BMJ*.

[27] Bellens M., Debien E., Claessens F., Gastmans C., Dierckx de Casterlé B. (2020). It is still intense and not unambiguous.” Nurses’ experiences in the euthanasia care process 15 years after legalisation. *Journal of Clinical Nursing*.

[28] Beuthin R., Bruce A., Scaia M. (2018). Medical assistance in dying (MAiD): Canadian nurses’ experiences. *Nursing Forum*.

[29] Bruce A., Beuthin R. (2020). Medically assisted dying in Canada: “beautiful death” is transforming nurses’ experiences of suffering. *Canadian Journal of Nursing Research*.

[30] Castelli Dransart D. A., Scozzari E., Voélin S. (2017). Stances on assisted suicide by health and social care professionals working with older persons in Switzerland. *Ethics & Behavior*.

[31] Denier Y., de Casterlé B. D., De Bal N., Gastmans C. (2009). Involvement of nurses in the euthanasia care process in flanders (Belgium): an exploration of two perspectives. *Journal of Palliative Care*.

[32] Denier Y., Dierckx de Casterlé B., De Bal N., Gastmans C. (2010). It’s intense, you know.” Nurses’ experiences in caring for patients requesting euthanasia. *Medicine, Healthcare & Philosophy*.

[33] Denier Y., Gastmans C., De Bal N., Dierckx de Casterlé B. (2010). Communication in nursing care for patients requesting euthanasia: a qualitative study. *Journal of Clinical Nursing*.

[34] Dierckx de Casterlé B., Denier Y., De Bal N., Gastmans C. (2010). Nursing care for patients requesting euthanasia in general hospitals in Flanders, Belgium. *Journal of Advanced Nursing*.

[35] Ho A., Joolaee S., Jameson K., Ng C. (2021). The seismic shift in end-of-life care: palliative care challenges in the era of medical assistance in dying. *Journal of Palliative Medicine*.

[36] Mills A., Wortzman R., Bean S., Selby D. (2020). Allied health care providers participating in medical assistance in dying: perceptions of support. *Journal of Hospice and Palliative Nursing*.

[37] Mills A., Bright K., Wortzman R., Bean S., Selby D. (2021). Medical Assistance in Dying and the Meaning of Care: Perspectives of Nurses, Pharmacists, and Social Workers. *Health*.

[38] van de Scheur A., van der Arend A. (1998). The role of nurses in euthanasia: a Dutch study. *Nursing Ethics*.

[39] Mathews J. J., Hausner D., Avery J., Hannon B., Zimmermann C., Al-Awamer A. (2021). Impact of Medical Assistance in Dying on palliative care: a qualitative study. *Palliative Medicine*.

[40] Pesut B., Thorne S., Schiller C., Greig M., Roussel J. (2020). The rocks and hard places of MAiD: a qualitative study of nursing practice in the context of legislated assisted death. *BMC Nursing*.

[41] Pesut B., Thorne S., Storch J., Chambaere K., Greig M., Burgess M. (2020). Riding an elephant: a qualitative study of nurses’ moral journeys in the context of Medical Assistance in Dying (MAiD). *Journal of Clinical Nursing*.

[42] Pesut B., Thorne S., Schiller C., Greig M., Roussel J., Tishelman C. (2020). Constructing good nursing practice for medical assistance in dying in Canada: an interpretive descriptive study. *Global Qualitative Nursing Research*.

[43] Oczkowski S. J., Crawshaw D., Austin P. (2021). How we can improve the quality of care for patients requesting medical assistance in dying: a qualitative study of health care providers. *Journal of Pain and Symptom Management*.

[44] Browning E. D., Cruz J. S. (2018). Reflective debriefing: a social work intervention addressing moral distress among ICU nurses. *Journal of Social Work in End-of-Life and Palliative Care*.

[45] Gabriel P. M., Smith K., Mullen-Fortino M., Ballinghoff J., Holland S., Cacchione P. Z. (2022). Systematic debriefing for critical events facilitates team dynamics, education, and process improvement. *Journal of Nursing Care Quality*.

[46] International Council of Nurses (2018). Evidence-based Safe Nurse Staffing. https://www.icn.ch/sites/default/files/inline-files/PS_C_%20Evidence%20based%20safe%20nurse%20staffing_1.pdf.

[47] Digby R., McDougall R., Gold M., Ko D., O’Driscoll L., Bucknall T. (2020). Introducing voluntary assisted dying: staff perspectives in an acute hospital. *International Journal of Health Policy and Management*.

[48] Cooper J., Wight J. (2014). Implementing a Buddy System in the Workplace: Paper Presented at PMI Global Congress 2014. https://www.pmi.org/learning/library/implementing-buddy-system-workplace-9376.

[49] Pesut B., Thorne S., Greig M., Fulton A., Janke R., Vis-Dunbar M. (2019). Ethical, policy, and practice implications of nurses’ experiences with assisted death: a synthesis. *Advances in Nursing Science*.

[50] Gitlin L., Hodgson N. (2016). Online training - can it prepare an eldercare workforce?. *Generations: Journal of the American Society on Aging*.

[51] Vermeer E., Devos T. (2021). The slippery slope syndrome. *Euthanasia: Searching for the Full Story*.

[52] Oh P.-J., Jeon K. D., Koh M. S. (2015). The effects of simulation-based learning using standardized patients in nursing students: a meta-analysis. *Nurse Education Today*.

[53] Arveklev S. H., Wigert H., Berg L., Burton B., Lepp M. (2015). The use and application of drama in nursing education—an integrative review of the literature. *Nurse Education Today*.

