# Supplementary material for: First-Hand Recommendations for Nursing Management to Support Nurses Involved in the Process of Hastened Death: A Systematic Review of the Qualitative Evidence
Source: J Nurs Manag. 2023 May 11;2023:8601814. doi: 10.1155/2023/8601814 (PMC11918919; doi:10.1155/2023/8601814)
Supplement: Supplementary Materials — Supplementary Table 1: inclusion and exclusion criteria. Supplementary Table 2: search strategy. Supplementary Table 3: synthesised findings, categories, findings, and corresponding illustrations. [file 8601814.f1.zip › Supplementary material_Table 1_Hastened death.docx]

**Supplementary material**

Table 1: Inclusion and Exclusion Criteria

| Criterion | Inclusion | Exclusion |
| --- | --- | --- |
| Population | Nurses experienced in caring for people requesting hastened death | Other professionals, nurses who did not yet care for people requesting hastened death; conscientious objectors |
| Phenomena | Personal experiences | attitudes |
| Publication | Peer review, qualitative studies | Quantitative studies, reviews, case reports, personal essays, poster, grey literature |
| Language | English, German | All other languages |
| Legislation | Hastened death legal at the time of the study | Hastened death illegal at the time of the study |
