# Supplementary material for: First-Hand Recommendations for Nursing Management to Support Nurses Involved in the Process of Hastened Death: A Systematic Review of the Qualitative Evidence
Source: J Nurs Manag. 2023 May 11;2023:8601814. doi: 10.1155/2023/8601814 (PMC11918919; doi:10.1155/2023/8601814)
Supplement: Supplementary Materials — Supplementary Table 1: inclusion and exclusion criteria. Supplementary Table 2: search strategy. Supplementary Table 3: synthesised findings, categories, findings, and corresponding illustrations. [file 8601814.f1.zip › Supplementary material_Table 2_Hastened death.docx]

**Supplementary material**

Table 2: Search Strategy

| # | Searchstring | Results |
| --- | --- | --- |
| CINAHL Plus via EBSCOhost | | |
| 1 | TX nurs* | 2,053,674 |
| 2 | experiences OR perceptions OR views OR feelings OR perspective | 1,078,877 |
| 3 | MH "Suicide, Assisted" OR MH "Euthanasia" | 6,566 |
| 4 | assisted suicide OR medical assistance in dying OR physician-assisted suicide OR physician assisted suicide OR assistance in dying OR assisted death OR euthanasia OR voluntary assisted dying OR right to die OR death with dignity | 16,964 |
| 5 | TX qualitative study OR qualitative research OR qualitative methods OR interview | 581,237 |
| 6 | S1 AND S2 AND (S3 OR S4) AND S5 AND S6; Filters: English, German | 843 |
| Medline via PubMed | | |
| 1 | nurs* | 1,068,837 |
| 2 | Nurses[Mesh] | 95,428 |
| 3 | experience* OR perception* OR view* OR feeling* OR perspective* | 2,482,062 |
| 4 | "assisted suicide" OR "medical assistance in dying" OR "physician-assisted suicide" OR "physician assisted suicide" OR "assistance in dying" OR "assisted death" OR euthanasia OR "voluntary assisted dying" OR “right to die” OR “death with dignity” | 33,652 |
| 5 | Euthanasia[Mesh] OR Suicide, Assisted[Mesh] | 18,554 |
| 6 | "qualitative study" OR "qualitative research" OR "qualitative methods" OR interview | 354,743 |
| 7 | (1 OR 2) AND 3 AND (4 OR 5) AND 6; Filters: English, German | 168 |
| Ovid | | |
| 1 | nurs* AND (experience* OR perception* OR view* OR feeling* OR perspective*) AND (assisted suicide OR medical assistance in dying OR physician-assisted suicide OR physician assisted suicide OR assistance in dying OR assisted death OR euthanasia OR voluntary assisted dying OR right to die OR death with dignity) AND (qualitative study OR qualitative research OR qualitative methods OR interview) | 109 |
| LIVIVO | | |
| 1 | nurs* AND (experiences OR perceptions OR views OR feelings OR perspective) AND (assisted suicide OR medical assistance in dying OR physician-assisted suicide OR physician assisted suicide OR assistance in dying OR assisted death OR euthanasia OR voluntary assisted dying OR right to die OR death with dignity) AND (qualitative study OR qualitative research OR qualitative methods OR interview); Filters: English, German | 158 |
| Web of Science | | |
| 1 | ALL=(nurs*) AND ALL=(experience* OR perception* OR view* OR feeling* OR perspective*) AND ALL=("assisted suicide" OR "medical assistance in dying" OR "physician-assisted suicide" OR "physician assisted suicide" OR "assistance in dying" OR "assisted death" OR euthanasia OR "voluntary assisted dying" OR “right to die” OR “death with dignity”) AND ALL=("qualitative study" OR "qualitative research" OR "qualitative methods" OR interview); Filters: English, German | 124 |
