# Supplementary material for: First-Hand Recommendations for Nursing Management to Support Nurses Involved in the Process of Hastened Death: A Systematic Review of the Qualitative Evidence
Source: J Nurs Manag. 2023 May 11;2023:8601814. doi: 10.1155/2023/8601814 (PMC11918919; doi:10.1155/2023/8601814)
Supplement: Supplementary Materials — Supplementary Table 1: inclusion and exclusion criteria. Supplementary Table 2: search strategy. Supplementary Table 3: synthesised findings, categories, findings, and corresponding illustrations. [file 8601814.f1.zip › Supplementary material_Table 3_Hastened death.docx]

**Supplementary material**

Table 3: Synthesised findings, categories, findings and corresponding illustrations

| Synthesised findings  (level 3) | Categories  (level 2) | Findings (examples)  (level 1) | Illustrations | Contributing studies |
| --- | --- | --- | --- | --- |
| If nurses are to provide good care, they need sufficient time resources. | Care for persons requesting hastened deaths is time consuming. | Expressing patience by taking time to listen carefully (UE); important that they could take the time that was needed in order to care for the patients and their relatives in a good way (E) | “Time is of essence for a good experience of the process. Can one make time for the patient and the family? Can one take time for oneself, for the team?” (Denier, Dierckx de Casterlé, et al., 2010, p. 44) | A6, A8 |
|  | Nurses face time restrictions. | Contextual limitations such as a lack of time (UE); preparation for a Medical Assistance in Dying intervention and how it indirectly consumed nursing resources (UE) | “…he comes over to die, and we haven't had any conversation with him, we didn't know him, was he married, did he have kids; the man just arrived for his injection, and that's it” (Bellens et al., 2020, p. 499) | A1, A4, A10 |
|  | Time constraints may have consequences. | being consumed by MAiD coordination while having less time for non-MAiD patients (UE) | ‘‘There was so much time sensitive coordination that my work ended up being much more focused on [MAiD] patients, at the expense of spending time with other patients and coordinating their care.’’ (Ho et al., 2021, p. 192) | A9, A13, A15 |
| If nurses are to provide good care, they need a supporting team. | Being part of a team and cooperation within a team is essential. | necessity of being part of a team (UE); teamwork as essential to a successful MAiD process (UE); relationships between those who provided palliative care and those who provided MAiD were becoming more congenial over time (UE); supportive relationships within the MAiD team were acknowledged as an integral part of the process of a successful MAiD provision (UE) | “So I don't know, I don't think it's something that I would be ready to do unless I felt that I was part of a team, [where] I felt there was good communication and good support.” (Beuthin et al., 2018, p. 516) | A1, A2, A6, A7, A9, A11–A16 |
|  | Debriefings are helpful to overcome uncertainty. | team debriefs were helpful in supporting staff by encouraging a welcoming space for different voices to come together and providing a platform for more conversations (UE) | ‘‘Debriefing is one of the most valuable and accessible. Those are so useful at reviewing cases that are highly relevant because of direct practice. They give us the opportunity to review what went well, what could have gone better and how we can do better next time.’’ (Ho et al., 2021, p. 192) | A9, A11, A16 |
|  | Senior nurses and managers play an important role for functioning teams. | especially attentive to the group dynamics of the nursing team (UE); importance of immediate special attention to the nursing team and physician (UE) | “And then we start to explore the team. And we notice that there are three kinds of nurses, three categories […] In the end we know very well how the group dynamics of the nursing team looks like. And that is very important.” (Dierckx de Casterlé et al., 2010, p. 2414) | A7, A8, A11, A15 |
| If nurses are to provide good care, they need clear guidelines and policies. | The caring process is complex, needs good organisation and develops over time. | good, practical organization of the process to be important (UE); complex and dynamic (UE); nothing must be overlooked (UE); practical organization of the euthanasia care process must be strong (UE); involvement in caring for patients requesting euthanasia develops over time (UE) | “I make sure that everything is well arranged for everyone involved: for the family, for the patient, for the physicians; for everyone at the bedside. It’s a bit of, well, yes — sort of coordinating everything. Making sure that everything proceeds well.” (Denier et al., 2009, p. 267) | A5–A8, A10,  A13–A15 |
|  | Clear guidelines and policies help to reduce uncertainty. | lack of clarity around legal and professional requirements for nurses (UE); risks of not providing good care or of running afoul of the legal system were just too great (UE) | “So, my big concern is if someone does approach me with a written request, what do I do from there? And I know the health region has developed no policies pertaining to what the process is.” (Pesut, Thorne, Schiller, Greig, & Roussel, 2020, p. 4) | A1, A2, A4, A5, A10, A13, A15 |
| If nurses are to provide good care, they need professional skills. | Caring for persons requesting hastened death is not routine nursing care and may lead to professional satisfaction. | expression of holistic nursing care (UE); providing comfort care, mitigating suffering, understanding of good death (UE); opportunity to do things differently (E); support nurses in their professional role (UE); more focused on trying to provide exactly the care that patients and their relatives need to make it through their last days together (UE); focus toward facilitating the most patient-centered death possible (UE); participating in MAiD positively reinforced their view of the profession (E) | “I find that the care changes once the decision to carry out euthanasia is taken. In the sense that, when the date is set, I have the impression that we respond to the patient’s wishes even more than usual. For instance, ‘I don’t want to be washed today’ or ‘I want to bathed seven times’, so to speak. I do have the impression that we comply with their requests with special and greater care than usual.” (Dierckx de Casterlé et al., 2010, p. 2415) | A1–A8, A10,  A12–A16 |
|  | Nurses must learn to distinguish between professional role and personal views. | keeping their personal views, feelings, and emotions to themselves (UE); nurses' personal beliefs did not have a role in practice (UE) | “It shouldn't matter what your personal values are. We have a moral and ethical obligation to put the patient at the centre of everything” (Pesut, Thorne, Storch, et al., 2020, p. 3877) | A4–A6, A13– A15 |
|  | There is a need for further training. | lack of mandatory education might contribute to routinizing death and cause unintended harm (E); recommended expanding MAiD education for PHCPs (UE) | “…they just did not know what they could say or do, and the lack of time for them to take the required education.” (Beuthin et al., 2018, p. 516) | A2, A3, A9, A15 |
| If nurses are to provide good care, they need personal skills. | Nurses have to respect the person and her/his decision. | showing respect for the patient as a person in the broad sense (UE); staying neutral and withholding judgment (UE); the right of patients to make that choice, but that did not mean they were comfortable participating (UE) | “if someone makes the choice, it's not whether they should or they shouldn't…if it's a personal decision and is best for them, then I think I should support it because it's not about me.” (Beuthin et al., 2018, p. 514) | A2–A5, A7, A8, A14, A16 |
|  | Nurses see themselves confronted with ethical challenges. | ethically challenging, despite following federal laws and patients’ wishes (UE); moral complexities of MAiD (UE) | ‘‘At the beginning I thought having more options, the better for people. What I’m realising is that sometimes having that option (…), causes suffering for the person passing away, for their loved ones, and for healthcare providers in general.’’ (Ho et al., 2021, p. 191) | A9, A10, A14 |
| If nurses are to provide good care, they need social skills. | Nurses must be able to establish relationships. | create a communicational atmosphere and to enter into personal relationships with patients and their family members (UE); establish relationships with patients and families quickly and skillfull (UE); family-centric care (UE) | “So, it encompasses engaging in a short period of time with a family and a client, developing a rapport very quickly and being very sensitive and intuitive about what kind of presence is wanted and what might be needed.” (Pesut, Thorne, Schiller, Greig, Roussel, & Tishelman, 2020, p. 6) | A4, A5,  A7–A10, A12, A13, A15 |
|  | Nurses act as advocates, guides, and supporters. | advocacy was frequently cited as a key component of care (UE); provide advice to the patient and the family (UE); simply to be there, supporting anyone who needs it (patient, family, colleagues, physician) (E) | “I think one of the biggest roles we can be as nurses are advocates, and advocating for what your patient wants isn’t necessarily what you would want for yourself. I think that’s something very important to remember. [. . .] So I think part of caring for your patient is really carrying out their directives for themselves too.” (Mills et al., 2021) | A2, A5,  A7–A9, A12, A15, A16 |
|  | Nurses must provide information. | provide the patient with all of the information necessary to make the right euthanasia decision before discussing non-euthanasia subjects with the patient (UE); supplying information voluntarily or upon request, in formal as well as informal situations (E) | “One of the things that I really have (emphasis) to do and that I find very important, is […] presenting alternative possibilities […] Also, providing information like: ‘This person will come and talk to you and that person too. We have to take these steps, followed by that, etc.’ All this happens in one, two or three conversations.” (Denier, Gastmans, et al., 2010, p. 3375) | A2, A5,  A7, A8,  A14–A16 |
|  | Nurses need communication competencies. | communication between the patient, the family and the nurses was considered an essential element (UE); reach a true understanding of the euthanasia request (UE); importance of having a voice in the decision-making process because they can provide specific information about the patient(E) | “They approach the topic in a roundabout way and then you have to clarify what they mean to ensure you are getting them the appropriate referral” (Pesut, Thorne, Schiller, Greig, Roussel, & Tishelman, 2020, p. 6) | A1, A2,  A5–A10, A13, A15, A16 |
| If nurses are to provide good care, they need to change their perspective. | The experience of a hastened death may differ from that of a natural death. | MAiD is changing [nurses’] view of dying overall (UE); felt that Medical Assistance in Dying negatively impacted the quality of death (E); more caring form of death (UE); feelings of unreality about a patient’s death (UE) | “With euthanasia, there is something very unreal, in the sense that someone, that a conscious person, goes in one or two minutes to death. So, from life to death. You see someone changing in his face; you see life that is pulling away. And that happens very quickly, and that is difficult to understand.” (Bellens et al., 2020, p. 496) | A1–A4,  A6, A8,  A10–A12, A15, A16 |
|  | Nurses’ understanding of the value of life may differ from that of the person requesting hastened death. | caring for a patient with a perception of life and quality of life that was not in accordance with their own was, for some nurses, a challenge (UE); determine where they personally stood in relation to the underlying values and practice of MAiD (UE) | “And if a patient who suffers psychologically asks you, ‘Come, give me a lethal injection, because it has been enough’. Well, I think, ‘Come on, man, do you really think life is worth so little? It is not that you are suffering from pain or something. This is time you take away from your family, your relatives’.” (Bellens et al., 2020, p. 498) | A1, A2, A4 |
|  | Nurses may be insecure about the request. | patients’ ambivalence regarding MAiD and challenges in managing families’ access inquiries on patients’ behalf (UE) | ‘‘There was a case where we weren’t sure who was advocating for MAiD—if it was the patient or her family who brought this as an option and pressured her.’’ (Ho et al., 2021, p. 191) | A3, A9, A14, A16 |
| If nurses are to provide good care, they must act as mediators. | …between the person requesting hastened death and the family | challenging conversations around supporting patients and resolving tension with families around Medical Assistance in Dying (UE) | “They [patients considering Medical Assistance in Dying] struggle.. . . [Medical Assistance in Dying patients say] I love my kids, how can I choose to leave them, you know. Those are big words. . .it’s hard to know how to respond to some of that.” (Mathews et al., 2021, p. 451) | A7, A8, A10 |
|  | …between the person requesting hastened death and the interprofessional team | between the patient and the physician, translate between both parties (E);  between the patient and the interprofessional healthcare team (UE) | “I can obviously advocate to the physician on behalf of that patient. . . patients say a lot of things to us nurses.” (Mills et al., 2021) | A6, A7, A12, A16 |
| If nurses are to provide good care, they need to know that there will be emotional ups and downs. | Caring for persons requesting hastened death is emotionally demanding and may come along with positive and negative feelings | overwhelming feelings, making the care process highly emotional and upsetting (UE); “exhausting” (UE); strong negative feelings and doubts (UE); mix of sometimes contradictory emotions (UE); feeling honored (UE); feeling happy and pleased for the patient (UE) | “And those are interactions that ‘drain’ you. You really are exhausted when you get out of there; you really have to recover from it.” (Bellens et al., 2020, p. 497) | A1–A6,  A9, A10,  A14–A16 |
|  | Nurses need help when deciding for or against participating in hastened death | colleagues were a source of moral influence when deciding whether to participate in MAiD (UE); what family might think about them extended to community members (UE) | “I've spoken to a number of clinicians who are much more experienced at this than I am and said to them, ‘You know, I didn't go into health care to end people's lives.’ And so, a couple of the leaders, who I have great respect for, said to me, ‘And nor did we, but, you know…’” (Pesut, Thorne, Storch, et al., 2020, p. 3874) | A14 |
|  | Nurses must develop coping strategies | various coping strategies intra as well as extra muros (UE); protective mechanisms such as treating assisted dying as a series of tasks (UE) | “My partner is my psychologist. I have a drink of Scotch, I don't sleep, I think about it the next day, I ruminate. I never forget these people and situations. I'm okay because I know that it's the right thing and I've relieved the suffering.” (Pesut, Thorne, Storch, et al., 2020, p. 3875) | A3, A6, A14 |
| If nurses are to provide good care, they need to know that hastened death will affect palliative care. | Nurses must be aware, that hastened death is a different approach to natural death | patients or patients' families could not accept the idea of a natural death (UE); natural process of dying seems to become less evident (UE) | ‘‘That is our experience: the natural process of dying won’t do any more. I regret that. I mean: we don’t need to give an injection to everyone.” (Denier, Dierckx de Casterlé, et al., 2010, p. 43) | A1, A6, A9 |
|  | Nurses struggle with structural shortcomings | media focused more on MAiD access than palliative/hospice care (E); inadequate accessibility to palliative care services for some patients in Canada (UE) | “But that our health care system contributes to suffering, and is doing nothing about our own contribution to that suffering, but then uses that very suffering to activate access to MAiD. It's absolutely ridiculous to me.” (Pesut, Thorne, Schiller, Greig, & Roussel, 2020, p. 6) | A9, A10, A13 |
|  | Nurses observe conflicts between palliative care and hastened death | withdrawal of services made it difficult for nurses to support good pain and symptom management while the patient was awaiting MAiD (UE); some patients did not want the trade-offs that might accompany such treatment (E) | “So we have a serious practice issue here. I’m mad as hell.” (Pesut, Thorne, Schiller, Greig, & Roussel, 2020, p. 5) | A10, A13, A15 |

Note: E = equivocal; MAiD = Medical Assistance in Dying; PHCP = Palliative health care professional; UE = unequivocal; US = unsupported
